# Co-creation of a complex, multicomponent rehabilitation intervention and feasibility trial protocol for the PostUraL tachycardia Syndrome Exercise (PULSE) study

**DOI:** 10.1186/s40814-023-01365-4

**Published:** 2023-08-15

**Authors:** Gemma Pearce, Nikki Holliday, Harbinder Sandhu, Helen Eftekhari, Julie Bruce, Emma Timms, Laura Ablett, Lesley Kavi, Jane Simmonds, Rebecca Evans, Paul Magee, Richard Powell, Shane Keogh, Gordon McGregor

**Affiliations:** 1 Coventry University, Coventry, UK; 2 University of Warwick, Coventry, UK; 3 University Hospitals Coventry and Warwickshire NHS Trust, Coventry, UK; 4 Patient and Public Involvement, Coventry University, Coventry, UK; 5 Oxford University Hospital NHS Foundation Trust, Oxford, UK; 6 PoTS UK, Wootton Wawen, UK; 7 University College London, London, UK; 8 Physioklinic, Kilcoole, Ireland

**Keywords:** Postural tachycardia syndrome, Exercise, Physical activity, Cardiac rehabilitation, Dysautonomia, Feasibility randomised controlled trial, Intervention development, Co-creation, Co-production, Patient and public involvement

## Abstract

**Background:**

There is a dearth of research to support the treatment of people with postural tachycardia syndrome (PoTS). Despite expert consensus suggesting exercise is recommended for this patient group, there are no randomised control trials examining this rigorously. The aim was to co-create a feasibility trial protocol and a rehabilitation intervention for people living with PoTS.

**Methods:**

The intervention and feasibility trial design were co-created as part of the PostUraL tachycardia Syndrome Exercise (PULSE) study. We used the ‘three co’s framework’ of co-define, co-design and co-refine. Recruitment included key national charities and National Health Service Trusts treating people living with PoTS in the UK. Eighteen patient and public involvement members attended the co-define session, and 16 co-creators with a mix of expertise attended the subsequent co-design and co-refine sessions. Seven intervention practitioners were trained in the rehabilitation intervention, providing feedback for further co-refinement.

**Results:**

The final co-created intervention comprises online physical activity, and lifestyle and behaviour change support sessions. It is based on functional movement activities using a patient-centred approach tailored to individual needs. Physical activity intensity is guided by individuals’ perception of effort rather than by objective measures. Recumbent bikes are provided for home use. Patients deemed randomisation to be acceptable because research in this area was considered important.

**Conclusions:**

An innovative approach was used to co-create the PULSE intervention and feasibility trial protocol to meet the evidence-based and logistical needs of people living with PoTS, clinicians, service deliverers, third-sector organisations, academics and funders. This can be used as a successful example and template for future research internationally. People living with PoTS were recognised as experts and involved in every aspect of conceptualisation, design and refinement. This complex rehabilitation intervention is currently being tested in a randomised feasibility trial comparing the PULSE intervention with best-practice usual care for people living with PoTS.

**Trial registration:**

ISRCTN45323485 was registered on April 7, 2020.

**Supplementary Information:**

The online version contains supplementary material available at 10.1186/s40814-023-01365-4.

## Background

Postural tachycardia syndrome (PoTS) is a clinical syndrome characterised by autonomic nervous system dysfunction resulting in an abnormal cardiovascular response to upright posture. PoTS is defined as a persistent heart rate increase of ≥ 30 beats per minute in adults when moving from a recumbent to a standing position; and the absence of orthostatic hypotension with associated symptoms for more than 3 months, not attributable to any other cause [1]. Symptoms include, but are not limited to, palpitations, light-headedness, pre-syncope and fatigue, varying in type and intensity for each individual [2]. PoTS can be debilitating, with persistent orthostatic intolerance significantly impacting activities of daily living [3] and quality of life [4]. Diagnosis and treatment pathways take longer for females than for males even though the PoTS demographic is predominantly female [5]. The prevalence of hypermobile Ehlers-Danlos syndrome (hEDS) and hypermobility spectrum disorders (HSD) is high in people living with PoTS, with 55% found to have hEDS or generalised joint hypermobility [6]. Further, approximately 20% have a diagnosis of chronic fatigue syndrome/myalgic encephalomyelitis (CFS/ME) [3]. The constellation of symptoms often impacts on exercise tolerance [7] leading to inactivity, which further exacerbates orthostatic intolerance, immobility and deconditioning [8, 9].

Exercise training is considered to have one of the strongest evidence bases among the existing treatment for PoTS, albeit rated as a ‘moderate’ level of evidence [1]. Expert consensus recommends ≥ 30-min non-upright exercise every other day, with a focus on aerobic reconditioning to potentially alleviate symptoms, improve quality of life and achieve remission in some patients [1]. These recommendations are based on four exercise training studies (total *n* = 191) demonstrating physiological improvement, reduced symptoms and improved quality of life [10–13]. Two studies (*n* = 25 [10] and 29 [13]) found that stroke volume and cardiac output were lower in people living with PoTS compared to healthy controls and that exercise improved this while decreasing heart rate. One study (*n* = 34) found that exercise improved upright hemodynamics, renal-adrenal responsiveness and quality of life compared to propranolol medication [11]. An observational PoTS registry study (*n* = 251) across seven countries, tested a community-based exercise and lifestyle intervention, reporting that symptoms and psychosocial morbidity improved in those completing the programme [12]. However, this study was limited by the lack of a control group and a high attrition rate of 59%. A recent quasi-experimental study (*n* = 77) found improved symptoms, cardiovascular function and quality of life with exercise when compared to control [14]. All five studies [10–14] were susceptible to selection bias, potentially excluding people with more severe symptoms of PoTS. Only one study mentions Ehlers-Danlos syndrome and only as an exclusion criterion [10].

There are no multicentre randomised control trials (RCT) testing exercise rehabilitation for people living with PoTS, and no studies employing intervention or research protocol co-development methodology. There is a lack of good quality evidence including people across the PoTS spectrum [15]. There is a risk that exercise may cause PoTS symptoms to worsen in the first 4–6 weeks before any lasting benefit is gained [16]. Therefore, cautious and well-designed clinical research is needed to examine the effectiveness of exercise rehabilitation for people living with PoTS.

To ensure relevance and applicability, it is vital that clinical research is co-produced with people who have, or are affected by, the specific medical condition being studied [17]. Co-production has largely been applied in qualitative research, but there is a strong rationale for this methodology to inform the design and implementation of interventions for testing in feasibility RCTs [18]. However, co-production is considered to be focused more on the implementation stage [19] and a component within the overarching concept of co-creation [20, 21]. Co-creation as an approach facilitates innovation with a wider range of stakeholders earlier in the development continuum, with the initiation of research ideas and iterative design [22, 23].

Underpinned by a participatory action research [24–26] and design thinking approach [27, 28], this project aimed to design and develop a complex rehabilitation intervention and feasibility trial protocol for people living with PoTS. This co-creation project was the first stage of the PostUraL tachycardia Syndrome Exercise (PULSE) study, with future stages of this research programme aiming to investigate the feasibility of conducting a multi-centre RCT testing the comprehensive co-created exercise rehabilitation intervention for people living with PoTS, compared to best practice usual care [29, 30].

### Overview of methods

**Fig. 1:**
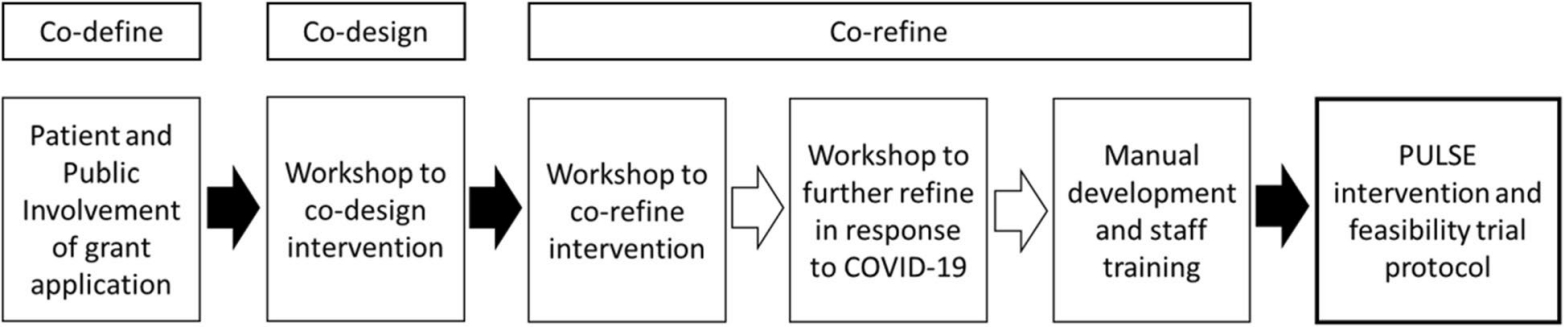
Overview of the development process for the feasibility trial protocol and intervention

The method was informed by the updated Medical Research Council guidance for the design of complex and contextual interventions that can contribute to system change in the real world [31]. The co-creative approach was based on the idea of developing collective creativity [20] using accessible methods to explore, communicate, reflect and document [32]. The co-creation methodology was underpinned by the three Co’s framework of co-define, co-design and co-refine (detailed in Fig. 1 and through the overarching structure of the methods and results sections) [23]. The co-define stage examines needs, strengths and resources; the co-design stage identifies and prioritises problems and formulates solutions for co-production; and the co-refine stage is the iterative and co-evaluative development from prototype to output with considerations of sustainability, dissemination and demonstrable change [23]. Guidance was used for reporting feasibility development [33] and maximising the use of qualitative approaches by embracing the iterative and dynamic nature of intervention development [34]. Detail relating to the template for intervention description and replication (TIDieR) [35], and the Consensus on Exercise Reporting Template (CERT) [36] is provided in the published protocols [29, 30]. This co-creation process adds novelty to that information by providing the underpinning reasons as to why and how decisions were made. The development of intervention components was guided by the overarching PRISMS (Practical Reviews In Self-Management Support) Taxonomy of Self-Management Support (detailed in the results of the co-define and co-refine sections) [37, 38]. Where behaviour change was relevant to a particular component, active ingredients were categorised using the Behaviour Change Techniques taxonomy [39] underpinned by the Capability Opportunity Motivation–Behaviour (COM-B) model [40] (detailed in the co-refine section).

Patient and public involvement (PPI) was of critical importance throughout the whole process of the feasibility trial protocol and intervention development, starting before the grant funding application was submitted, and continuing with PPI co-applicants on the grant funding application, in the co-creation workshops, and as part of the core trial management group. PPI members from these different activities were involved in decision making, proof-reading of the funding application and study documents, and authorship of this paper. This included decisions made about research questions, priorities, outcome measures, inclusion criteria, recruitment, and participant burden. People living with PoTS across the spectrum of symptoms and severity were invited throughout, including people with related conditions, such as hEDS/HSD and CFS/ME. The methods and results of the iterative process are described in order throughout this section, and Additional file 1 summarises feedback and responses received during the development process.

### Co-define: Patient and public involvement (PPI) teleconference

#### Methods

To inform the grant application, two PPI focus groups, recruited publicly through social media, were carried out funded by a West Midlands Research Design Service PPI Grant. Focus groups took place via teleconference with a total of 18 adults with PoTS and those who support them, such as partners or parents. Teleconference was requested by PPI members to allow the inclusion of people living with PoTS across the UK, including those who were bedbound and/or struggled to travel. People who were unable to attend or had additional comments after the focus group were invited to feedback by email. Discussions addressed issues relating to the research need, appropriate exercise, barriers to being active, terminology, recruitment, inclusion/exclusion, randomisation, outcomes, and the relevant components and delivery of self-management support interventions underpinned by the PRISMS Taxonomy of Self-Management Support [38, 37]. Two members of this group also provided feedback on the written research grant funding application and became members of the trial management group after funding was secured from the British Heart Foundation.

#### Results

Overall, the research topic was seen to be an important area of study but there was a key concern around using the language of ‘exercise’ and avoiding vigorous levels of activity. In response to this, the term ‘physical activity’ will be used in this publication in preference to ‘exercise’. The PPI members suggested that the focus should be on functional movement-based activities with slow progression, addressing lifestyle and behaviour change using a patient-centred approach tailored to people’s needs. PPI members felt happy for physical activity sessions to be delivered in a supervised rehabilitation centre first prior to participants learning to continue independently. Recumbent physical activity was preferred to minimise the impact of orthostatic intolerance. Although swimming is often used in clinical rehabilitation programmes due to lower weight-bearing demands, caution was advised for people living with PoTS because of the potential for exacerbation of symptoms resulting from heat and humidity, and the rapid transition back to weight-bearing activity after swimming.

It was important to our PPI groups that physical activity be sustainable and ultimately manageable within daily routines. Pacing and other psychological techniques to help the maintenance of the behaviour were viewed as important alongside the physical activity intervention. Care was advised in the application of psychological support techniques as people living with PoTS are often told incorrectly that ‘it is all in their head’ [41]. Engaging in physical activity in a specialised cardiac rehabilitation setting was viewed as a positive because this could avoid the potential embarrassment of experiencing symptoms in ‘normal’ exercise environments, such as near syncope or collapse, and trained professionals would supervise the sessions. Cardiac rehabilitation centres, however, are often associated with use by older adults, whilst people living with PoTS indicated a preference for dedicated sessions for younger people. PPI members were concerned about the potential lack of knowledge among rehabilitation professionals in relation to comorbidities such as hEDS/HSD and CFS/ME where inappropriate support can exacerbate symptoms. It was agreed that practitioners would benefit from specialist training in these areas.

The PPI group confirmed they would accept randomisation in a trial, even though this would mean they may not receive the intervention, because it was felt that the research was important and necessary. The outcome measures considered to be important in a PoTS trial were PoTS symptoms, activities of daily living, and heart rate. The PPI group agreed from the discussions that the main components of the PULSE intervention from the PRISMS Taxonomy of Self-Management Support [38, 37] were ‘lifestyle advice and support’ related to physical activity; ‘social support’ through peer physical activity, lifestyle and behaviour change support sessions; and ‘training/rehearsal for psychological strategies’ supporting physical activity engagement. The PPI members’ preferred delivery model was a mixture of group and one-to-one sessions supervised by healthcare professionals who listened to people living with PoTS and understood the everyday challenges faced.

### Co-design: Face to face workshop

#### Methods

The British Heart Foundation grant included funding for the co-design, co-production and co-refinement of the intervention and feasibility trial protocol with a range of key stakeholders. The co-design phase aimed to engage PoTS stakeholders (co-creators): a total of 16 people participated alongside three facilitators. Expertise amongst the co-creators included people living with PoTS, clinical exercise physiologists, academics/researchers, health psychologists, nurses, charity representatives, healthcare professionals delivering PoTS care in National Health Service (NHS) Trusts (West Midlands, London), a physiotherapist, a patient services coordinator and a clinical trial coordinator (with some co-creators offering multiple expertise, such as a researcher with PoTS and a cardiac advanced nurse practitioner with PoTS). Facilitators were designers, project managers and experts in co-creation facilitation. The workshop was held in the neutral space of a sport’s ground conference centre to facilitate an environment of equal voice and reduce feelings of hierarchy that academic or hospital settings can provide [23]. The facilitators’ task was to maintain momentum, manage group tasks and support discussion, prioritising equal voice amongst co-creators, and not to act with power as leaders of the session.

The co-design workshop began with introductions, rules of engagement, and an ice-breaker activity. The goals of the project, limitations and objectives of the project were explained. The next activity used the co-creative CUbe tool (Coventry University CUbe) [42–44] to explore concerns raised in the co-define PPI sessions. Co-creators were split into groups, with each group focusing on a specific concern (worries about the intervention, words or language that might put people off participating in the feasibility study, etc.). Each group was provided with a cardboard CUbe on which they could write their thoughts and feelings regarding the intervention. At the end of the activity, each group fed back to the room to enable wider discussion.

Subsequently, the focus of the workshop moved towards the design of specific aspects of the intervention using the Carousel method [45]. The co-creation team was presented with an outline of the ‘prototype’ PULSE intervention and research protocol, fixed to the wall on large paper. Each aspect of the research protocol and intervention (research participant recruitment, intervention, outcome measures) was presented and explained by the facilitators. Co-creators were split into three groups, with each group asked to work on a different aspect of the research and intervention. Questions to guide the co-creators’ thoughts were included on the wall alongside the displayed intervention plans. The co-creation team was provided with pens, ‘post-it’ notes and stickers on which to write their thoughts and affix to the wall. Facilitators worked with the groups to ensure that the task was understood and to provide prompts if required. After 15 min, the groups were asked to stop and move on to the next aspect of the intervention to ensure that every co-creator had a chance to impact upon the research protocol and intervention. At the end of the task, a facilitator summarised the comments and feedback.

#### Results

A full list of the changes made to the draft intervention and research protocol is presented in Additional file 1. Regarding recruitment to a feasibility trial, it was suggested that participants could be identified from local secondary care records and specialist PoTS clinics. Co-creators further identified that recruitment could be supported via charities, social media and private physiotherapy practices. In terms of inclusion and exclusion criteria for a trial, co-creators discussed that those also diagnosed with hEDS/HSD (or an older similar version of these syndromic hypermobility diagnoses) and CFS/ME should be included, but those with mitochondrial disease should be excluded. The original draft of the intervention suggested that those with mental health issues may be excluded as this could prevent engagement with trial procedures. However, co-creators questioned if this was appropriate and argued that the exclusion criteria for mental health needed to be better defined as many conditions would not necessarily preclude engagement. Intervention staff would need to be trained by specialists to individualise physical activity sessions to accommodate symptoms, severity and comorbidities as required.

With regard to the intervention itself, co-creators suggested that two to three sessions of physical activity per week would be daunting, so proposed that this be reduced, or tailored specifically to the individual. Co-creators stressed that it was important to have the same practitioner delivering the intervention throughout. There was discussion as to whether heart rate and blood pressure should be measured throughout the intervention—PoTS patients preferred to judge progress subjectively by how they were feeling overall, rather than objectively with heart rate and blood pressure. Psycho-social support sessions were agreed to be important; however, it was decided to refer to these as ‘lifestyle and behaviour change support sessions’ to avoid the participants’ concern that PoTS symptoms may be perceived to be ‘all in their head’ [41]. Online and home-based physical activity sessions were suggested to allow flexibility. However, because the funding was for an in-person and on-site intervention, it was agreed that this should be the focus of the initial feasibility trial, with the idea that online tools could be developed to compliment the intervention at a later stage.

For the outcome measures, it was suggested that too many were proposed (Orthostatic Hypotension Questionnaire, EQ-5D-5L, Fatigue Impact Scale, Short Physical Performance Battery, symptoms experienced during physical activity, adverse events). Patients stated that a large number of questionnaires was not advisable with the potential for ‘brain-fog’ symptoms in PoTS, which could lead to incomplete data collection. PoTS patients were keen to avoid the use of the ‘tilt table test’ as a physical outcome measure, as this was often a very uncomfortable and distressing procedure. Informed by these results, a draft feasibility trial protocol and rehabilitation intervention were co-produced for refinement in the next workshop.

### Co-refine 1: Online workshop

#### Methods

Co-creators from the first workshop (*n* = 11 and four facilitators) were invited to take part in a second workshop hosted online, to co-refine the draft co-produced intervention and trial protocol before finalisation. ‘Big Blue Button’ software was used to present slides in a semi-structured framework summarising the trial and intervention plan. Co-creators could directly interact and annotate the slides live, write thoughts in the chat box or say what they thought as part of a focus group-style discussion. This started with an icebreaker to familiarise co-creators with the technology by asking them to put a pin on a map and say where they were joining from. Co-creators were then asked to comment on inclusion and exclusion criteria, feasibility outcome measures, the intervention itself and the language used to describe the intervention.

#### Results

Broadly, co-creators agreed with the inclusion and exclusion criteria for the trial. It was agreed that people experiencing chronic fatigue should be included, as long as fatigue was carefully monitored and managed during the trial, with particular attention to identifying and avoiding post-exertional malaise [46]. It was also confirmed that being a wheelchair user would not exclude someone from participating, as they would still be able to take part in the physical activity intervention, designed to be flexible and adapted to individual needs. Feasibility outcomes were agreed, for example the number of participants recruited, willingness of clinicians to support recruitment, and adherence to the intervention.

With regard to the intervention itself, there was further discussion about whether people living with PoTS would be happy to attend existing gym-based physical activity groups (for example, cardiac rehabilitation groups). Views were mixed, with some co-creators thinking that this would be acceptable if the other members of the group also had a long-term condition. Participants were to be informed if the groups were to be mixed-sex. However, others felt that ensuring there were other people with a PoTS diagnosis in the group would increase acceptability and thus adherence to the trial. The physical activity intervention is described in more detail elsewhere [29]. Briefly, physical activity will be undertaken in a controlled gym environment with progression staged in response to participant tolerance and symptoms. Moderate-intensity dynamic cardiovascular exercise will be prescribed depending upon ability. In addition, ‘functional fitness training’ should aim to improve orthostatic tolerance and general musculoskeletal deconditioning. Physical activity should be versatile and individualised, incorporating cardiovascular and functional resistance training components.

With regard to physical outcome assessments, there was concern that the inclusion of the ‘active stand’ test may be off-putting. However, it was also argued that being unable to complete the test due to anxiety, symptom severity or physical fitness levels would be a measurement in itself. Following this co-refinement session, the feasibility trial protocol was finalised for submission to NHS and Coventry University ethical approval processes (changes to the protocol and intervention from this stage are summarised in Additional file 1) and published [29].

### Co-refine 2: online workshop in response to COVID-19 guidelines

#### Methods

The COVID-19 pandemic, which instigated restrictions on movement and ‘social mixing’ in the UK intermittently throughout 2020 and 2021, meant that the intervention as originally funded and co-designed, with in-person, centre-based delivery, was no longer possible. To address this issue, an additional online co-refine session was conducted to discuss the adaptation of the original intervention and trial protocol [29] to adhere to COVID-19 guidelines. In total 11 co-creators took part, alongside 4 facilitators. This used the same Big Blue Button as the previous online workshop and started with an icebreaker asking co-creators to annotate pictures of activities that they may have done during the COVID-19 lockdown. Then, a redesigned live online trial protocol was proposed and developed based on discussions from previous co-design sessions.

#### Results

During this second co-refine session, it was agreed to deliver the PULSE intervention as a structured home-based physical activity programme (published as an updated protocol) [30]. Briefly, the programme will be supported by a participant manual and pre-recorded online content. The participants will use functional (weight/chair-based, including exercise ball and band) activities and equipment (recumbent bike) and attend supervised live online group sessions. Online videos were added to the intervention providing asynchronous physical activity guidance to participants. This included low-, medium- and high-intensity sessions with accommodations for participants with lower mobility or functional capacity covering a spectrum of symptoms, severity and comorbidities, including hEDS/HSD and CFS/ME. Ethical approval was granted for the revisions and a summary of the agreed changes can be found in Additional file 1.

### Co-refine 3: Manual development, intervention staff training and feedback

#### Methods: manual development

By consolidating all the data gathered from the previous workshops, the physical activity and psychosocial intervention components were mapped [47, 48] alongside the PRISMS Taxonomy of Self-Management Support [37, 38], behaviour change theory and the Behaviour Change Techniques taxonomy [39, 49] to develop a fully manualised intervention (Table 1). The overarching theories used to inform the development of the intervention were behaviour change theory (COM-B) [40], social learning [50] and group-based learning [51]. Practitioner and participant workbooks were developed as a guide and a comprehensive tool to consolidate learning. These were based on manuals from other rehabilitation trials [46–48] and circulated to the wider intervention team to incorporate feedback on content and layout.

**Table 1:** Intervention components, PRISMS Taxonomy of Self-Management Support, theoretical underpinnings and Behaviour Change Technique taxonomy

Overarching category	Intervention component and/or delivery	Aims	PRISMS Taxonomy of Self-Management Support component	Theoretical framework(s)	Behaviour Change Techniques taxonomy
Specific delivery mode	Online one-to-one sessions Initial consultation and check-ins	To develop practitioner-participant relationship and to assess medical physical and psychosocial barriers and facilitators to physical activity participation	Training/rehearsal for psychological strategies Practical support with adherence (behavioural)	Bio-psychosocial, COM-B	Focus on past success Self-talk
	Group based sessions	To share experiences and learning and offer social support	Social support	Social learning, COM-B, group-based learning	Feedback on behaviour Behavioural practice/rehearsal
Physical activity	Supervised online live group physical activity sessions Bands and balls were provided to all participants to support these sessions	To guide, support and educate participants on appropriate, safe and tailored physical activity, as well as how and when to progress To provide guidance, practical skills and motivation to improve: Movement and Mobility Stamina Muscular strength Autonomic control Cardiorespiratory fitness Flexibility Agility Co-ordination Balance Proprioception	Lifestyle advice and support Practical support with adherence (behavioural) Provision of equipment	COM-B Self-regulation theory	Instruction on how to perform a behaviour Demonstration of the Behaviour Exposure Graded tasks Body changes Adding objects to the environment
	On-demand library of physical activity sessions Asynchronous online resource Bands and balls were provided to all participants to support these sessions	To encourage maintenance and sustainability of physical activity behaviour external from supervised sessions	Lifestyle advice and support Practical support with adherence (behavioural) Provision of equipment	COM-B Self-regulation theory	Instruction on how to perform a behaviour Demonstration of the Behaviour Exposure Graded tasks Body changes Adding objects to the environment
	Recumbent exercise bike at home independently	To provide equipment for participants to use at home	Provision of equipment	COM-B Self-regulation theory	Adding objects to the environment
Lifestyle and behaviour change support sessions tailored to a physical activity focus	Expectations, motivation	To introduce intervention framework and allow participants to reflect on their own motivations and expectations	Training/rehearsal for psychological strategies	Bio-Psychosocial, COM-B, Acceptance Theory	Information about health consequences Pros and cons
	Goal setting and planning	To increase understanding of PoTS, impact and allow participants to set their own realistic targets and practice planning	Training/rehearsal for psychological strategies	Bio-Psychosocial, COM-B	Goal setting (behaviour) Goal setting (outcome) Action planning
	Fear avoidance and breaking the cycle	To introduce the concept and relationship between anxiety, fear and impact on daily activities including movement. To explore strategies to break this cycle such as pacing of activities	Training/rehearsal for psychological strategies	Bio-Psychosocial, COM-B Fear-avoidance model	Problem-solving Behavioural practice/rehearsal Framing/reframing
	Emotional impact (stress and mood)	To introduce and increase understanding of stress and unhelpful thoughts, emotional impact and effective coping strategies	Training/rehearsal for psychological strategies	Bio-Psychosocial, COM-B CBT principles	Reduce negative emotions Information about social and environmental consequences Monitoring of emotional consequences
	Sustainable behaviour change and working through setbacks	To reflect on learning throughout the programme, progress made, future planning and enhance confidence to sustain behaviour change	Training/rehearsal for psychological strategies	Social learning, COM-B, group-based learning	Review behaviour goal(s) Comparative imagining of future outcomes
Guidance provided	Participant workbook	To give participants information related to each part of the intervention, summary pages, case studies and questions to answer to help progress through the intervention. This provides behavioural adherence support and a behaviour diary	Practical Support with adherence (behavioural)	COM-B	Self-monitoring of Behaviour Habit formation Habit reversal Generalisation of a target behaviour
	Practitioner manual	To offer practitioners a step-by-step guide to deliver the intervention	Not applicable because the Taxonomy is focused on components to support the person with the condition only	COM-B Motivational interviewing	Credible source Verbal persuasion about capability

#### Results: manual development

In addition to the original three identified PRISMS taxonomy components following the PPI co-define activities (‘lifestyle advice and support’, ‘social support’ and ‘training/rehearsal for psychological strategies’), two additional taxonomy components were included in the intervention: ‘provision of equipment’ and ‘practical Support with adherence (behavioural)’) [37, 38]. It is also worth noting that as part of the feasibility trial protocol, participants could make contact if they had a question or concern arising from their participation in the intervention. This, in itself, may provide interventional benefits in relation to the ‘provision of easy access to advice or support when needed’ component, even though not officially part of the intervention.

Group sessions were designed to promote connection and are particularly important to help with social support for anyone feeling isolated or stigmatised by living with PoTS [52]. Group learning and support promote learning through shared experiences and modeling of behaviour (adapting coping strategies to enhance wellbeing). Self-efficacy and confidence can also be enhanced through observing others, following instruction or demonstration that was incorporated into the PULSE intervention.

The COM-B [40] mapped three core principles of the intervention: capability, opportunity and motivation. To enhance participants perceived capability (psychological and physical), lifestyle and behaviour change support sessions were designed to enhance understanding of PoTS and physical activity in relation to cognitions (unhelpful thinking), mood and emotions, such as fear avoidance of physical activity. The aim was to integrate physical activity sessions with the lifestyle and behaviour change support sessions to increase confidence and skills through supervised practice and over time increase levels of physical activity.

To address factors associated with opportunity, the intervention was designed to allow access to resources such as a workbook with information about the intervention structure, aims and content of all sessions, and access to equipment if needed. In addition, the lifestyle and behaviour change support sessions were designed to explore social norms, as co-creators described living with PoTS as isolating and were acutely aware of the lack of understanding from some of those around them, often including family, friends and clinicians.

**Fig. 2:**
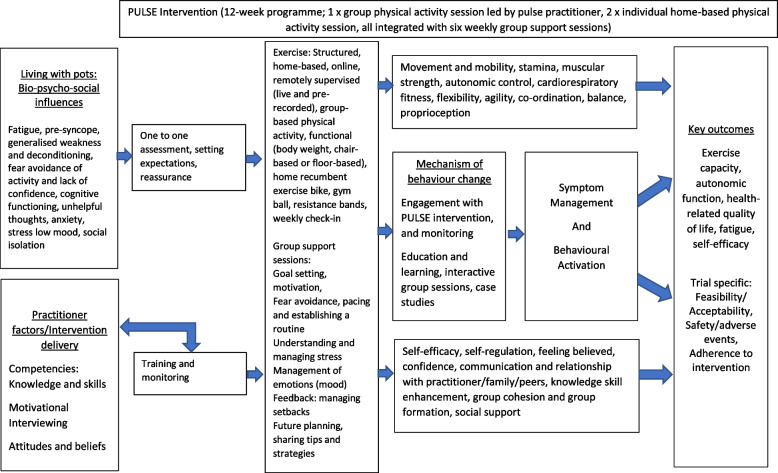
PULSE logic model

To improve motivation, the lifestyle and behaviour change support sessions included case studies and discussion topics exploring beliefs around PoTS and physical activity management strategies, values, goals, sense of self-identity and perceived ability to engage in the programme and beyond, whilst gaining awareness of current barriers and facilitators that may be preventing behaviour change such as fear, stress and anxiety (worry about the future). The biopsychosocial influences of living with PoTS allowed us to address the physical, emotional and cognitive factors identified in the literature and through our co-creation work. This is demonstrated in a logic model (Fig. 2).

Feedback from the intervention team included ensuring the integration between the physical activity, and lifestyle and behaviour change support session topics was clear by incorporating information linking the different topics, especially relating to the impact of PoTS on daily living. For the practitioner workbook, feedback was to include questions that directed the conversation with specific cues around the topic area, because each section had a limited time, and having too many generic questions could be difficult to manage during the sessions.

#### Methods: intervention staff training and feedback

Training sessions were held face-to-face at Coventry University and online with seven clinical exercise physiologists trained to be PULSE intervention practitioners. Training covered PoTS (clinical presentation, symptoms and impact), physical activity assessment and prescription, motivational interviewing and communication skills. Competencies required to deliver the intervention included the ability to recognise and support the management of PoTS symptoms with communication and reassurance. Post-pandemic, each intervention facilitator was given additional training online on the adapted manual and workbook.

#### Results: intervention staff training and feedback

Feedback from training included (1) more practice was required to feel confident in delivering the lifestyle and behaviour change support sessions; (2) to incorporate fear avoidance early on in the programme to allow participants time to adapt their thinking, action their goals and encourage engagement with the physical activity sessions; (3) to simplify the thought diary to capture unhelpful thoughts; and (4) to add in case studies where possible to allow exploration of the topic.

### Final PULSE intervention for feasibility testing

**Fig. 3:**
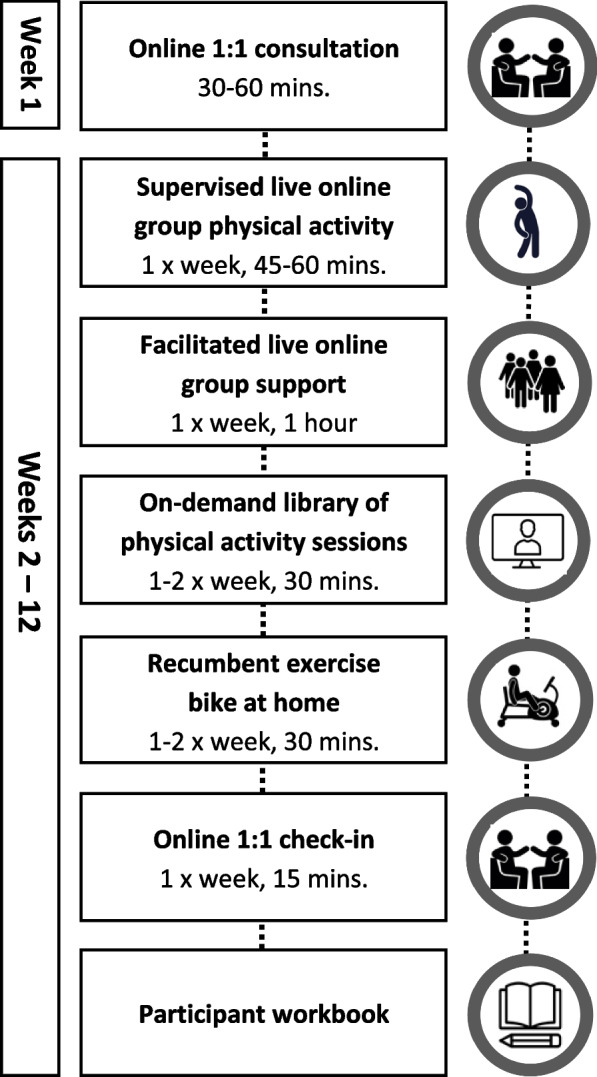
Final format and components of the ‘PostUraL orthostatic tachycardia Syndrome Exercise’ (PULSE) intervention. Note: The timings provided in Fig. 3 are aims for the participants but may not be feasible to achieve initially. The level and time will therefore be tailored to their needs during intervention delivery

The final PULSE intervention consists of (1) an online 1:1 consultation with a PULSE practitioner, (2) 12 weeks of supervised live online group physical activity, (3) six facilitated live online group lifestyle and behaviour change support sessions, (4) on-demand library of physical activity sessions, (5) recumbent exercise bike at home, (6) weekly online 1:1 check-in and (7) participant workbook (Fig. 3).

#### Quality assurance

Quality assurance of delivery of the PULSE intervention will be assessed through observations by a Health Psychologist, with feedback given to each practitioner. Quality assurance will assess delivery as well as adherence to the practitioner manual, structure and topics. During the PULSE intervention training, practitioners were informed that the manual was a guide and that there was some flexibility, but the aim should be to deliver all the content and cover all the topics.

## Discussion

We co-created a complex, theory-based and multicomponent intervention incorporating physical activity, lifestyle and behavioural support for people living with PoTS, to be tested within a randomised feasibility trial. This innovative, transparent and systematic approach showcases best practices as a successful example of how to fully apply iterative and person-centred co-creative processes to optimise intervention and feasibility trial protocol development. PPI was engaged throughout the development process alongside other key stakeholders, as part of the decision-making core team and co-creation activities. This study provides a success story of overcoming key barriers to PPI addressing the current international vision for research for 2025 [53]. Public awareness and communication were increased through the public research engagement hub of ‘hEDS together’ (www.hEDStogether.com) and the related charities of PoTS UK and Syncope Trust And Reflex Anoxic Seizures (STARS). Resources and PPI payment were secured through the West Midlands Research Design Service PPI fund and the British Heart Foundation grant was written specifically to include the iterative processes of co-creating the intervention and feasibility protocol. Recognition included acknowledgment on the PULSE (www.pulse-project.coventry.ac.uk) and hEDS Together websites throughout the project where consent was given and invitation to co-author publications. Consistency was enhanced by using the three co’s framework of co-creation [23], and leadership of the PPI was by a researcher with PoTS and experience of being a PPI member for other research (GP), working alongside a multidisciplinary team.

There has been concern over the potential for physical activity interventions to cause harm for populations impacted by chronic fatigue [46], so it was especially important that from the outset this intervention and protocol were developed with and for people living with PoTS. PoTS is thought to be an underdiagnosed condition and people living with PoTS often report not feeling listened to by healthcare professionals [8]. It was a priority to ensure that this group was recognised as experts in their condition and that their voices were considered as equal, if not more important, than other team members. An area where our PPI reported a difference to some current literature [54] was in relation to swimming as an advisable physical activity for people living with PoTS. While this might be suitable for some, our PPI group was more cautious of the after-effects of swimming. This study was more than PPI, using a broader co-creative approach from project initiation with a range of stakeholders as co-creators working together. Co-creative approaches can help to align research, service development and clinical commissioning, therefore increasing the impact of research findings [20]. With human experience at the core, considering the whole system and it’s interrelationships within a co-creative approach helps to ensure that conflict and power were utilised positively [20]. Using the three co’s framework of co-creation [23] allowed an in-depth examination of living with PoTS and ensured a range of important voices were engaged in the decision-making processes throughout conception, design and refinement of the research. Involving people with a range of expertise, context and experience enables potential barriers to be overcome during the process and increases the chance of success and impact [23]. This co-creation framework is recommended for use in future intervention or service development where co-production and PPI principles should underpin this process. People living with PoTS involved in the co-creation of the PULSE trial and intervention, presented with a range of symptom severity, from those who were bedbound to those who were able to do physical activity in their daily lives. Research in PoTS is currently limited and would benefit from the inclusion of a wider range of ethnicities, genders and cultures. The next step of this programme of research is to use a mixed methods approach to test the feasibility of conducting a randomised controlled trial comparing the PULSE intervention with best-practice usual care for people living with POTS. This will be the first PoTS intervention to have been co-created, explicitly based in the UK, and tailored to include people with a range of symptom severity and comorbidities.

### Supplementary Information

**Additional file 1.** Feedback and responses throughout the development process of the feasibility trial protocol and intervention.

## Data Availability

The data analysed during the current study are available from the corresponding author on reasonable request.

## Abbreviations

CERT: Consensus on Exercise Reporting Template
CFS/ME: Chronic fatigue syndrome/myalgic encephalomyelitis
COM-B: Capability opportunity motivation—behaviour model
PoTS: Postural tachycardia syndrome
NHS: National Health Service
PPI: Patient and public involvement
PULSE: PostUraL tachycardia Syndrome Exercise (PULSE) study
STARS: Syncope Trust And Reflex Anoxic Seizures
RCT: Randomised control trial
TIDieR: Template for Intervention Description and Replication
UK: United Kingdom
