# Supplementary material for: Co-creation of a complex, multicomponent rehabilitation intervention and feasibility trial protocol for the PostUraL tachycardia Syndrome Exercise (PULSE) study
Source: Pilot Feasibility Stud. 2023 Aug 15;9:143. doi: 10.1186/s40814-023-01365-4 (PMC10426060; doi:10.1186/s40814-023-01365-4)
Supplement: Supplementary file 1 — Additional file 1. Feedback and responses throughout the development process of the feasibility trial protocol and intervention. [file 40814_2023_1365_MOESM1_ESM.docx]

**Additional file** **1: Feedback and responses throughout the development process of the feasibility trial protocol and intervention**

| **Stage** | **Feedback and considerations** | **Response** |
| --- | --- | --- |
| Co-define: PPI | Although swimming is often used in rehabilitation because of the lower weight bearing aspect, caution was placed for people with PoTS because of the heat and humidity. Changing rapidly back to weight bearing afterwards can exacerbate symptoms. It was important to our PPI groups, that exercise be sustainable and ultimately manageable within daily routines. | Removed swimming as a suggested exercise in the grant application and replaced with recumbent exercise. |
|  | There was a key concern around using the language of ‘exercise’ and avoiding vigorous levels of activity. It was suggested that the focus should be on movement-based activities with slow progression using a patient-centred approach tailored to people’s needs. | Ensured tailored approach of functional movement-based activities. Intervention deliverers trained on the needs and concerns of people with PoTS in relation to ‘exercise’ and referred more to as physical activity. |
|  | PPI determined that some people with PoTS may not be comfortable attending existing cardiac rehabilitation sessions, for reasons of age (i.e. younger PoTS patients felt uncomfortable in exercise classes with older people), gender (i.e., mainly women rather than men) and embarrassment about symptoms (i.e., pre-syncope / syncope). | Added to grant explaining that dedicated sessions will be provided if necessary, and this will be evaluated as a feasibility criterion. |
|  | The importance of psychological support in PoTS was a very prominent theme from our PPI work. PoTS can be associated with significant psychological and social consequences from lack of PoTS knowledge and awareness amongst health care practitioners and social support networks. These may amplify the condition. Comprehensive behavioural change and motivational strategies to improve adherence and compliance to exercise are needed. | Every second week, before or after exercise, participants will receive a one-to-one 30-minute behavioural and motivational session delivered by a PULSE practitioner, with the aim of improving short and long-term adherence to exercise. |
|  | The PPI groups were cautious of fatigue but felt they would be willing to still do the exercise programme knowing that it might get worse before it gets better (as long as it doesn’t just make it worse). | Ensured that this was listed as a potential issue in participant information sheets but looked to reduce the possibility of this as much as possible by focusing on moving more with tailored activities and progression to support the individual’s needs. |
|  | For those with hypermobility in our PPI group, there was anxiety about the lack of knowledge amongst exercise professionals. Not supporting people with this condition appropriately can exacerbate associated pain or potential dislocations, and thus specialist training was considered vital. | PULSE practitioners were trained by a hypermobility specialist professional on safe and effective lower limb and core stability exercise with particular attention to range of movement, exercise tempo, and pacing. |
|  | Our PPI groups advised that the offer of four weeks of supervised exercise (twice/week) after completion of the trial follow-up period, for those in the control arm, would be an incentive, however, they would still be prepared to be randomised even if this was not available because they felt this research was highly important. | Randomisation and a control arm were included in the feasibility study. |
| Co-design: face to face workshop | **Inclusion criteria feedback and recommendations:**   - Participants must have a formal diagnosis of PoTS - Able to attend a centre up to 2-3 times a week for exercise training - Include hypermobility (e.g., hEDS/HSD and similar diagnosis), Chronic Fatigue Syndrome (CFS)/Myalgic Encephalomyelitis (ME) - Refine inclusion/exclusion criteria regarding mental health, and define mental health issues that will affect whether or not people are able to engage. | **Inclusion criteria included in protocol:**   - Participants will have a formal clinician’s diagnosis of PoTS made at each centre - Able to attend a centre 1-2 times per week for physical activity programme 8-12 weeks - Include CFS/ME, hEDS/HSD and similar previous syndromic hypermobility diagnoses. Intervention staff to be trained by specialists to individualise physical activity sessions to accommodate symptoms, severity and comorbidities as required. - Anxiety/depression - People on all types of medication for PoTS or other conditions (as long as it does not increase risk of exercise) |
|  | **Exclusion criteria feedback and recommendations:**   - Exclude mitochondrial disease - Need to be clearer about contraindications, are they specific to PoTS or general to physical activity? - Unable to travel to centres for exercise training - Have previously been randomised into a group for this trial but have since dropped out - Pregnancy | **Exclusion criteria included in protocol:**   - Exclude mitochondrial disease - Contraindications will be anything that poses a serious risk to health during exercise according to existing guidelines for clinical exercise - Current high levels of structured exercise - Unable to travel to and attend a centre - Serious mental health/cognitive issues that could increase the risk of exercise - Previously randomised - Pregnancy |
|  | **Outcome measures feedback and recommendations**   - Record number of patients (screened, eligible, recruited, randomised, withdrawn, retained) - Willingness of clinicians to support recruitment - Adherence to exercise intervention - Length of time it takes people to complete patient-based outcome measures - Willingness of people to join non-PoTS exercise rehabilitation programmes - Acceptability - Final patient-based outcome for the definitive trial (how will this be decided)? - Might only expect patients to complete 3 of the proposed questionnaires max, - Some physical tests are not enjoyable (e.g. tilt table test), cause anxiety and can increase symptoms for days. Need to be clear about risks of the outcome measures and remind participants of their right to withdraw. - There are too many questionnaires for patients to complete – questionnaires and brain fog make multiple long questionnaires difficult to complete. Some of these measures might worry people. - 10-minute stand test is difficult for most PoTS patients, can the patients stop at any time? Can the length of time they stand be used as a measure? Patients need to know they won’t be jeopardising the trial by not completing certain tests. - Can we include a questionnaire which include changes in confidence and quality of life? - Monitor people’s symptoms during and after exercise - Include COMPASS dysautonomia questionnaire - Remove orthostatic hypotension scale as this is validated in 65-year old+ patients with low blood pressure who may have multi-morbidities, it is not specific to the PoTS patient group as not everybody with PoTS has low blood pressure. - The EQ-5D-5L is a good measure – quality of life is important - Daily fatigue scale is a good measure | **Outcome measures will include:**   - Number of patients (screened, eligible, recruited, randomised, withdrawn, retained) - Willingness of clinicians to support recruitment - Adherence to exercise intervention – sessions attended and successfully completed - What physical and questionnaire outcome measures people are prepared to do*   *Physical and questionnaire outcomes will include:   - 10-minute stand now optional - Short physical performance battery (optional, or can complete certain parts of the test only) - Six-minute walk - Health status (EQ-5D-5L) - Fatigue impact scale - Generalised self-efficacy scale - COMPASS dysautonomia questionnaire (changed from orthostatic hypotension scale) - Continuous heart rate monitoring during exercise added - Symptoms experienced during physical activity - Adverse events – dislocations/injuries, increased pain/fatigue, exacerbations, fainting   Things we couldn’t change for the outcome measures included the general feasibility outcomes, reducing the overall number of questionnaires and we needed to include one physical outcome. However, participants will not be forced to complete these outcome measures, and we will record how many people were able to successfully complete to inform the definitive trial. |
|  | **The intervention – feedback and recommendations**   - Concerns regarding the use of word ‘exercise’ – the word can be daunting, and we need to be clear about what we mean by exercise. - 2-3 sessions a week may be daunting for patients, reduce to 1-2 times per week, or have a set number of sessions over a 12-weeks period. - Would there be the possibility of having online exercise sessions for some patients? - PoTS patients would prefer to judge their physical activity levels by how they feel, rather than by physical measures such as heart rate - Patients will need flexibility on how long it takes to familiarise themselves with the exercises to begin doing them at home - Concerns that it is difficult to define what is “moderate intensity” - There was a questions of whether people would have access to specialised equipment at home? - Meeting others with PoTS and hearing positive experiences of exercising with PoTS is useful - Psychosocial and motivational support elements will be useful, but concern of calling it psychosocial because of the stigma people with PoTS can experience of symptoms being ‘all in their head’. | **The intervention will include:**   - Supervised physical activity in a cardiac rehab centre, 1-2 times per week for 8-12 weeks - Physical activity intensity not regulated by heart rate (although this will be recorded for research purposes) - Intensity dictated by individual tolerance, rather than aiming for “moderate intensity”. Focus on breathlessness and rating of perceived exertion - Introduce home exercises as soon as people are ready (changed) - Use mentors/advocates to promote the benefits of physical activity - Do not use the word exercise – focus on physical activity - Flexible and adaptable physical activity prescriptions to suit individual needs - Psychosocial and motivational support to remain included but called ‘lifestyle and behaviour change support sessions’ - The funding was for an in-person intervention at the centres and so physical activity sessions could not be done online for this study. There was not a budget to loan exercise equipment for use at home. |
| Co-refine 1: online workshop | Broadly participants agreed with the inclusion and exclusion criteria of the trial, but highlights that people with chronic fatigue need to be carefully monitored, particularly with regards post-exertional malaise.  Participants sought confirmation that wheelchair users would not be excluded  With regards the intervention itself, there were mixed views regarding whether or not people with PoTS would be comfortable attending existing exercise groups, e.g. cardiac rehabilitation groups, although it was considered that this may be acceptable as long as the other members of the groups also had a long term health condition.  Feasibility outcomes were agreed  There were concerns that the active stand test would uncomfortable and/or off-putting but it was agreed that being unable to complete the test due to anxiety or physical fitness levels would be a measure for the feasibility trial in itself. | As per previous intervention, adverse events including increased pain and fatigue/exacerbations of symptoms will be recorded and dealt with appropriately  It was confirmed that wheelchair users would not be excluded due to the flexible nature of the intervention.  It will be made clear to participants that they will be attending groups with people who may have long term health conditions other than PoTS  No changes were required to the feasibility outcomes  Active stand test to remain an outcome measure, although participants are not required to complete it. |
| Co-refine 2: workshop responding to COVID-19 | Existing online programmes for people with COPD, heart failure, angina, and ischaemic heart disease were considered and adapted to suit the PoTS population, with broad agreement on the new proposals from the co-creation participants. | Decision made with approval from funder to change the delivery of the PULSE intervention to a structured home-based physical activity programme supported by a participant manual, and live and pre-recorded online content using functional (weight/chair based, including use of exercise ball and band) exercises and equipment (recumbent bike) with additional remote supervised sessions.  Funding was therefore moved from the original travel budget to support the purchasing of recumbent bikes, balls and bands for trial participants to use at home.  Online videos were added to the intervention providing asynchronous physical activity guidance to participants. This included low, medium and high intensity sessions with accommodations for participants with lower mobility or functional capacity covering a spectrum of symptoms, severity and comorbidities, including hEDS/HSD and CFS/ME. |
| Co-refine 3: manual development and staff training | The manual had an aim of outlining each session, with clear objectives and questions to facilitate discussions and consolidate learning for participants. Feedback for the manual from the wider intervention team included the following points:  To ensure the topics allowed exploration of the impact of PoTs and not just outlining general stress or unhelpful behaviours. | This was amended in the manual and specific examples of understanding stress and stress management used examples of the impact of living with PoTs. The same was provided for challenging unhelpful behaviours specifically related to participant experiences. |
|  | There was also a suggestion to keep the questions focused to aims of the programme including specific reference to the physical activity sessions rather than generic. Feedback suggested this would help facilitate discussions on PoTs, and make best use of the time within each session. | The questions were amended throughout the manual to reflect this. |
|  | Thought diary: There was a suggestion to add a PoTs related example for participants which could help them complete it for themselves. | The thought diary was amended with a specific PoTs related example. |
|  | Training: the feedback from the training included having more examples and support to be able to deliver the group lifestyle and behaviour change support sessions. | Further support was scheduled for the facilitator who would deliver the intervention, with regular contact and refresher training as required. |
